# 2276 Development of the expanded access oversight committee at Michigan Medicine

**DOI:** 10.1017/cts.2018.281

**Published:** 2018-11-21

**Authors:** Kevin Weatherwax, Ray Hutchinson, George Mashour, Misty Gravelin

**Affiliations:** University of Michigan School of Medicine

## Abstract

OBJECTIVES/SPECIFIC AIMS: Expanded Access is an avenue for patients with no available treatment options to access investigational drugs and devices for clinical therapy. This process requires physicians treating these patients to submit requests to the FDA and the local IRB, processes which are typically unfamiliar to clinicians. METHODS/STUDY POPULATION: With the goal of reducing burden and ensuring access to investigational products, Michigan Medicine established the Expanded Access Oversight Committee in January 2015. This committee brought together key stakeholders to develop appropriate policy and infrastructure to support these requests. RESULTS/ANTICIPATED RESULTS: Outcomes from this committee have resulted in a uniform process with a single point of entry for interested physicians and patients. With standardized policy implemented across the institution, a revised IRB application has been developed that is more tailored to Expanded Access and an informed consent document has been developed specific to the clinical use of investigational therapies. To ensure timely execution of these agreements, the contracts office has streamlined the process for negotiating Expanded Access agreements with manufacturing companies. Further development has begun with the Michigan Clinical Research Unit to provide space for clinical visits in Expanded Access cases, allowing for initiation of outpatient therapy. These changes have allowed Michigan Medicine to support triple the number of Expanded Access requests, including more than 45 Expanded Access requests in fiscal year 2018. DISCUSSION/SIGNIFICANCE OF IMPACT: Institutional support for Expanded Access requests within a large academic medical center is feasible and can increase access to investigational therapies bfor patients.

